# Alendronate use and bone mineral density gains in women with moderate-severe (stages 3B–5) chronic kidney disease: an open cohort multivariable and propensity score analysis from Funen, Denmark

**DOI:** 10.1007/s11657-020-00746-z

**Published:** 2020-06-01

**Authors:** M. Sanni Ali, Martin Ernst, Danielle E. Robinson, Fergus Caskey, Nigel K. Arden, Yoav Ben-Shlomo, Mads Nybo, Katrine H. Rubin, Andrew Judge, Cyrus Cooper, M. K. Javaid, Anne P. Hermann, Daniel Prieto-Alhambra

**Affiliations:** 1 Faculty of Epidemiology and Population Health, Department of Non-communicable Disease Epidemiology, London School of Hygiene and Tropical Medicine, London, UK; 2 Center for Statistics in Medicine, Nuffield Department of Orthopaedics, Rheumatology and Musculoskeletal Sciences, University of Oxford, Oxford, UK; 3 Department of Public Health, Saint Paul Hospital Millennium Medical College, Addis Ababa, Ethiopia; 4 OPEN, Department of Health, University of Southern Denmark, Odense, Denmark; 5 Department of Public Health, Clinical Pharmacology and Pharmacy, University of Southern Denmark, Odense, Denmark; 6 Population Health Sciences, Bristol Medical School, University of Bristol, Bristol, UK; 7 Nuffield Department of Orthopaedics, Rheumatology and Musculoskeletal Sciences, University of Oxford, Oxford, UK; 8 MRC Lifecourse Epidemiology Unit, Southampton, UK; 9 Department of Clinical Biochemistry and Pharmacology, Odense University Hospital, Odense, Denmark; 10 Department of Endocrinology, Odense University Hospital, Odense, Denmark; 11 GREMPAL Research Group (Idiap Jordi Gol Primary Care Research Institute) and CIBERFes, Universitat Autonoma de Barcelona, Barcelona, Spain

**Keywords:** Alendronate, Bone mineral density, Chronic kidney disease, Osteoporosis, Incidence density sampling, Propensity score

## Abstract

**Summary:**

Bisphosphonates are contraindicated in moderate-to-severe chronic kidney disease patients. However, they are used to prevent fragility fractures in patients with impaired kidney function, despite a lack of evidence on their effects on bone density in these patients. We demonstrated that Alendronate had a positive effect on bone in these patients.

**Purpose:**

This study aimed to assess the association between alendronate use and bone mineral density (BMD) change in subjects with moderate-severe chronic kidney disease (CKD).

**Methods:**

We created a cohort of CKD stage 3B–5 patients by linking all DXA-based measurements in the Funen area, Denmark, to biochemistry, national health registries and filled prescriptions. Exposure was dispensation of alendronate and the outcome was annualized percentage change in BMD at the femoral neck, total hip and lumbar spine. Individuals were followed from first BMD to the latest of subsequent DXA measurements. Alendronate non-users were identified using incidence density sampling and matched groups were created using propensity scores. Linear regression was used to estimate average differences in the annualized BMD.

**Results:**

Use of alendronate was rare in this group of patients: propensity score matching (PSM) resulted in 71 alendronate users and 142 non-users with stage 3B–5 CKD (as in the 1 year before DXA). Whilst alendronate users gained an average 1.07% femoral neck BMD per year, non-users lost an average of 1.59% per annum. The PSM mean differences in annualized BMD were + 2.65% (1.32%, 3.99%), + 3.01% (1.74%, 4.28%) and + 2.12% (0.98%, 3.25%) at the femoral neck, total hip and spine BMD, respectively, all in favour of alendronate users.

**Conclusion:**

In a real-world cohort of women with stage 3B–5 CKD, use of alendronate appears associated with a significant improvement of 2–3% per year in the femoral neck, total hip and spine BMD. More data are needed on the anti-fracture effectiveness and safety of bisphosphonate therapy in moderate-severe CKD.

## Introduction

Chronic kidney disease (CKD) is a multi-factorial disease associated with a range of metabolic diseases and complications. The prevalence of these complications increases rapidly in patients with stage 3B, an estimation of glomerular filtration rate (eGFR) below 45 ml/min/1.73m^2^ (1). It is also an independent risk factor for osteoporosis. More than one in four people with osteoporosis have moderate or severe CKD (2). CKD has been shown to predict not only low bone mass due to accelerated bone loss (3) but also fracture risk, with a doubled risk in patients with stage 3 CKD (eGFR between 30 and 59 ml/min/1.73 m2) (4); a 2.5- to 3-fold risk in those with stage 3B CKD (4); and a four-times higher fracture incidence amongst patients with stage 4 CKD (eGFR between 15 and 29 ml/min/1.73 m2) (5, 6) or in renal replacement therapy (7, 8).

Osteoporosis may develop in patients with a reduced eGFR due to age-related decrease in renal function or in patients with intrinsic renal disease (1). Patients with CKD due to intrinsic renal disease often have additional risk factors for osteoporosis such as glucocorticoid use, hyperparathyroidism, and vitamin D deficiency (9). Bone mass or bone mineral density (BMD) is a recognized key determinant of future fracture risk in postmenopausal women (10) and patients with CKD, next to bone microarchitecture, bone geometry and rate of turnover (11). Measurement of BMD at the lumbar spine, femur neck or total hip by dual-energy X-ray absorptiometry (DEXA) is the standard method for diagnosis of osteoporosis and evaluating fracture risk, particularly in non-CKD patients. However, its significance and interpretation in CKD patients have been controversial due to a combination of CKD-induced changes in bone and mineral metabolism (1). Recently, several studies have shown that BMD is a good predictor of fracture risk in CKD stages 3–5; as a result, the 2017 Kidney Disease Improving Global Outcomes (KDIGO) guideline recommended systematic BMD measurement in CKD patients (12–14). BMD measurement is often assessed before anti-fracture treatment decisions are made; this is in part due to the fact that therapeutic trials in osteoporosis usually require a low BMD value as an entry criterion. Hence, medications are often licensed for use in patients below a given BMD threshold. In addition, BMD is also monitored as a surrogate marker to evaluate the response of an individual patient to treatment as well as the efficacy of a treatment in clinical trials (15–18).

The most important clinical goal of osteoporosis treatment is the reduction in fracture risk, due to high morbidity and mortality associated with it, especially in elderly people. Whilst there are effective therapies to reduce the risk of osteoporotic fracture, the use of oral bisphosphonates (i.e. first-line anti-osteoporosis therapies) is restricted in patients with CKD for several reasons. First, there are safety concerns related to the risk of bisphosphonates worsening kidney function and other adverse events which are already increased in patients with CKD such as severe hypocalcaemia or hyperphosphatemia, or acute kidney injury (9). Second, given the biological mechanism of how CKD weakens bone differs from osteoporosis, it is far from established that bisphosphonates will have a similar beneficial effect in improving BMD. Importantly, participants in these trials with CKD are likely to be healthier and have less comorbidities compared with patients with CKD in the real-world setting. This leaves moderate-severe CKD patients with a very high fracture risk effectively untreatable. In addition, the relationship between changes in BMD during treatment with oral bisphosphonates and fracture risk is still less clear since the numbers of patients with moderate or severe CKD recruited for the pivotal trials were low (15–17, 19–21). However, clinical trial participants with higher BMD gains on oral bisphosphonate therapy had a significantly lower risk of fracture than participants with no change or a reduction in BMD (20). Hence, BMD is an accepted surrogate marker in bridging studies of anti-osteoporosis treatments (22) to test different dosing regimens (23) and efficacy in other populations such as men (24) and in patients taking glucocorticoids (25).

The relationship between BMD change and fracture risk in postmenopausal osteoporosis versus bone fragility in CKD may differ given the different aetiopathology of bone fragility in CKD (26) with particular reference to potential adynamic bone disease. The aim of this study was to examine the association between bisphosphonate use and changes in BMD over time in subjects with moderate-to-severe CKD (stages 3B, 4 and 5).

## Methods

### Data source

We used the Odense University Hospital Database (OUHD) which holds DXA-measured BMD data for the whole of the Funen region, the second-largest island of Denmark, comprising 36,024 individuals examined between August 1990 and February 2015. Serum creatinine is part of the routine panel of blood tests performed at the Odense University Hospital as part of osteoporosis care. Primary care practices and hospitals in this Danish region use the same clinical biochemistry laboratory, making all biochemistry values (including serum creatinine tests) available for this study. We were able to calculate the eGFR for participants in this cohort using the CKD-EPI formula (27). Pharmacy drug dispensations of primary and secondary care prescriptions can be tracked back to 1995 from the Danish Prescriptions Register and linked using unique national patient identifiers to OUHD. Pharmacy dispensations are recorded using Anatomic Therapeutic Classification (ATC) codes.

The Open Patient Data Exploratory Network (OPEN) provides a unique platform for linking clinical, dispensation and biochemistry data. It is an approved Statistics Denmark institutional partner for linking to diagnoses and comorbidity recorded using ICD-10 codes in Danish hospital records. The Network has previously been used to link clinical biochemistry to fracture outcomes in numerous studies (28–32). Ad hoc extraction and linking to renal function from the Odense University Hospital clinical biochemistry database was done as part of this study using a similar approach. In the OUHD, 30% of individuals examined (over 10,000 subjects) were recommended osteoporosis treatment, with alendronate as the first-line drug. In Denmark, reimbursement for alendronate and other alendronates requires a DXA assessment or an X-ray verified spine or hip fracture. The study was approved by the Data Protection Agency (OUH 15/37999) and by Statistics Denmark (ref. 705079). All statistical analyses were done on de-identified patient data merged at Statistics Denmark.

### Study participants and design

All patients registered in OUHD aged 40 years or older at the age of biochemistry testing, with at least one eGFR measurement below 45 ml/min/1.73 m^2^ (CKD stages 3B, 4 and 5), at least 2 years of follow-up data available and at least two DXA BMD measurements recorded 2 or more years apart were eligible for inclusion. Previous users of any anti-osteoporosis medication (except calcium and vitamin D supplements) in the year before eGFR testing and those with more than 1 year between the closest renal measurement (eGFR) and DXA (BMD measurement) were excluded. The first dispensation of alendronate (therapy initiation) was considered the index date for alendronates users. Participants, users of alendronate, were risk-set-matched with replacement to non-users on ± 5 years of the year of birth at index date, meaning a non-user could be matched to several alendronate users. For every alendronate user, up to five non-users at index date were randomly selected from a large pool of non-users. Selection bias was thus minimized whilst preserving statistical power, as initial non-users could become alendronate users and will be included later as users.

### Exposure, outcome and confounders

Alendronate use, defined by pharmacy dispensations in the Danish Prescriptions Register, was the main exposure. Users of alendronates were identified using pre-specified lists of ATC codes. Like prescriptions, dispensation data do not necessarily reflect treatment duration. Treatment episodes were created using a previously validated algorithm that accounts for non-adherence (or non-compliance) and defines treatment episodes of continuous exposure. Any overlapping prescriptions of the same drug were interpreted as an early collection of a repeat prescription. Any overlapping days between two prescriptions of the same drug were added to the end of the period covered by the two prescriptions. Any two prescriptions of the same drug were concatenated and considered as one prescription if the gap between the end of the first prescription and the start of the second prescription was less than 30 days.

The primary study outcome was annualized femoral neck BMD percentage change, relative to the previous or baseline BMD, up to 3 years after alendronate use (or non-use), measured using a DXA. If more than two measurements were available, the last DXA measurement 2–3 years from the index date was considered. Any measurements within 1 year between each other were excluded, as they were unlikely to provide clinically relevant information. Secondary outcomes were similarly defined as annualized BMD percentage changes, measured at the lumbar spine and total hip. If participants had serial spine measurements, we used the vertebrae that were still assessable at the last recorded visit to calculate the rate of BMD change. A sensitivity analysis used DXA measurements taken 3–5.5 years after the index date, since change in BMD has been shown to plateau after 3 years (33).

Potential confounders were pre-identified using clinical knowledge and a literature review. Confounders were measured at baseline and included age, sex, BMI, baseline eGFR, fracture history, comorbidities (Charlson index, renal disease and diabetes), hospital contacts in the previous year and concomitant drug use (number of ATC in the year before index, aromatase inhibitors, antihypertensives, antidepressants and antidiabetics). As systemic steroids have an effect on BMD and are commonly used in certain renal conditions, we accounted for these drugs in a more granular fashion. We calculated the number of defined daily doses in the previous year and stratified into quartiles.

### Statistical analyses

In a primary analysis, propensity scores for alendronate use were estimated using multivariable logistic regression, including the confounders (Table 1). Interactions between age and BMI as well as age and baseline eGFR were added to the propensity score equation to improve the balance of the baseline characteristics. The calculated propensity scores were used to match each alendronate user with to up to five comparable non-users with a calliper width of at most 0.02 SDs on the logit of the propensity scores. Balance before and after matching was checked using the absolute standardized mean difference (ASMD). An ASMD of 0.10 or less was considered acceptable and representative of a good balance.

**Table 1:** Baseline characteristics for eligible and included bisphosphonate users

	Eligible	Included
Characteristic		
Unique patients (*N*)	270	71
eGFR, mean (SD)	37.2 (7.2)	37.9 (8.0)
Dead within 3 years of first bisphosphonate	35 (13.0%)	0 (0.0%)
Age, median (IQR)	82 (76–87)	79 (70–83)
Gender, female *N* (%)	268 (99.3%)	71 (100.0%)
Index BMD^a^, mean (SD)		
Total hip	0.657 (0.123)	0.678 (0.117)
Femoral neck	0.551 (0.110)	0.560 (0.106)
Spine	0.791 (0.165)	0.782 (0.166)
Fractures, *N* (%)		
Hip	21 (7.8%)	5 (7.0%)
Non-hip other	21 (7.8%)	5 (7.0%)
Non-hip osteoporotic	28 (10.4%)	6 (8.5%)
Charlson score, *N* (%)		
0	142 (52.6%)	39 (54.9%)
1	64 (23.7%)	18 (25.4%)
2	29 (10.7%)	7 (9.9%)
≥ 3	35 (13.0%)	7 (9.9%)
Concomitant drugs, *N* (%)		
Systemic corticosteroids	98 (36.3%)	29 (40.8%)
Anticoagulants	163 (60.4%)	35 (49.3%)
Statins	106 (39.3%)	30 (42.3%)
Antidiabetics	38 (14.1%)	7 (9.9%)
Antihypertensives	198 (73.3%)	58 (81.7%)

^a^Up to 1 year before index date

*BMD*, bone mineral density; *discont.*, discontinued; *eGFR*, estimated glomerular filtration rate; *IQR*, interquartile range; *OP*, osteoporotic; *SD*, standard deviation

Linear regression modelling was used to estimate average differences (expressed as beta coefficient and 95% confidence intervals) in the annualized BMD change (per cent versus index measure) at the femoral neck (primary outcome), total hip and lumbar spine (secondary outcomes). Two post hoc analyses were conducted to deal with the low number of alendronate users eligible for inclusion in the final analyses:

(i) A linear regression model including all eligible alendronate users and non-users and adjusted for the previously calculated propensity scores
(ii) A multivariable linear regression model, adjusting for the same set of confounders included in the propensity score model, was fitted to model annualized percentage BMD change according to alendronate use, with 95% confidence intervals.

All analyses were conducted using R (34) and STATA 15 (35). Statistical syntax was programmed at Oxford using mock data extracted from the analytical dataset. These programs were run ‘in situ’ by a statistician at Odense, Denmark. Final results were verified by two of the authors (MSA and DPA).

## Results

### Participants and baseline characteristics

**Fig. 1:**
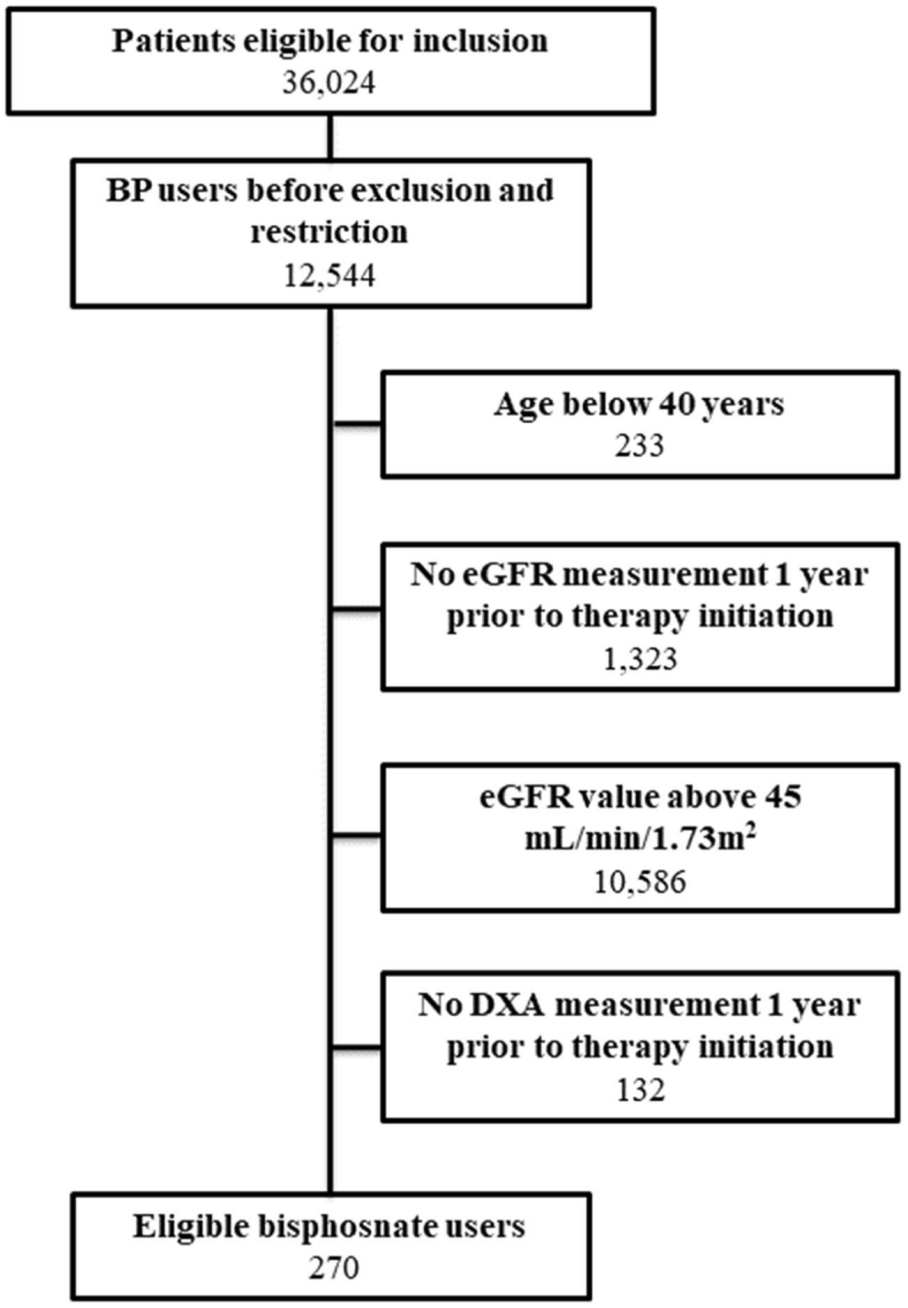
Construction of treatment episodes based on prescription data. Most of the eligible patients were on alendronate (264/270, 98%)

We identified 36,024 patients in the linked OUHD BMD database who were eligible for the study, including 12,544 (34.8%) patients who were alendronate users within the study period. We excluded 233 (1.9%) who were younger than 40 years at the time of therapy initiation, 1323/12,544 (10.5%) who did not have an eGFR measurement recorded in the previous year and 10,586/12,544 (84.4%) who had an eGFR value of 45 or above. Of the remaining 402 eligible alendronate users, a third (132/402, 32.8%) had no DXA measurement in the year before therapy initiation. Therefore, 270 alendronate users (264, 98% on alendronate) were eligible for the study (Fig. 1). Amongst these, 35 (13.0%) died during follow-up, 93 (34.4%) discontinued alendronate therapy before having a repeat BMD and 142 (52.6%) did not have a repeat BMD between 1 and 3.5 years after the first scan after therapy started. We, therefore, included 71 alendronate users in the study. In parallel, 23,713 non-users were initially available for comparison. Amongst these, 1228 (5.2%) died during follow-up. Only 1609 (6.8%) had repeat BMD measures between 1 and 3.5 years after their index DXA and were eligible for the analysis.

Baseline characteristics for all 270 eligible alendronate users and the final sample of 71 are reported in Table 1. As expected, participants included in the final analyses were younger (79 verses 82 years old on average), healthier (54.9% versus 52.6% with a Charlson score of 0) and more likely to use systemic corticosteroids (40.8% versus 36.3%), statins (42.3% versus 39.3%) and antihypertensives (81.7% versus 73.3%) than those excluded.

Table 2 reports baseline characteristics for the included participants, stratified by alendronate use. Both groups only included women and had an average age of 79–80 years. As expected, alendronate users had a much lower index BMD (0.56 versus 0.62 g/cm^2^ at the femoral neck), were more likely to use systemic corticosteroids (40.8% versus 32.8) and had a much higher prevalence of the previous fracture (7.0% versus 1.7% for hip fracture history) than non-users. Low bone mass, prevention of glucocorticoid-induced osteoporosis and secondary fracture prevention are the most likely indicators of alendronates in patients with moderate-to-severe CKD. All of these variables and the confounders listed in Table 1 are adjusted for using propensity score and multivariable regression models.

**Table 2:** Baseline characteristics according to bisphosphonate use

	Before matching		After matching	
	Users	Non-users	Users	Non-users
Patients (*N*)	71	1492^a^	40	142
eGFR, mean (SD)	37.9 (8.0)	36.1 (7.7)	37.1 (9.1)	38.0 (6.7)
Age, median (IQR)	79 (70–83)	80 (76–85)	79 (75–82)	79 (73–84)
Gender, *N* (%)				
Females	71 (100%)	1492 (100%)	40 (100.0%)	142 (100.0%)
Males	0 (0%)	0 (0%)	0 (0%)	0 (0%)
Bone mineral density				
Total hip	0.678 (0.117)	0.754 (0.144)	0.663 (0.119)	0.738 (0.134)
Femoral neck	0.560 (0.106)	0.627 (0.121)	0.547 (0.104)	0.617 (0.104)
Spine	0.782 (0.166)	0.877 (0.171)	0.806 (0.146)	0.881 (0.176)
Fractures, *N* (%)				
Hip	5 (7.0%)	28 (1.9%)	5 (12.5%)	(*n* < 5)
Non-hip other	5 (7.0%)	99 (6.6%)	(*n* < 5)	(*n* < 5)
Non-hip osteoporotic	6 (8.5%)	89 (6.0%)	(*n* < 5)	6 (4.2%)
Charlson score, *N* (%)				
0	39 (54.9%)	778 (52.1%)	27 (67.5%)	95 (66.9%)
1	18 (25.4%)	322 (21.6%)	8 (20.0%)	23 (16.2%)
2	7 (9.9%)	213 (14.3%)	(*n* < 5)	9 (6.3%)
≥ 3	7 (9.9%)	179 (12.0%)	(*n* < 5)	8 (5.6%)
Drugs, *N* (%)				
Systemic corticosteroids	29 (40.8%)	493 (33.0%)	11 (27.5%)	51 (35.9%)
Anticoagulants	35 (49.3%)	818 (54.8%)	18 (45.0%)	61 (43.0%)
Statins	30 (42.3%)	522 (35.0%)	17 (42.5%)	51 (35.9%)
Antidiabetics	7 (9.9%)	316 (21.2%)	5 (12.5%)	26 (18.3%)
Antihypertensives	58 (81.7%)	1.331 (75.8%)	32 (80.0%)	112 (78.9%)
Additional diagnoses, *N* (%)				
Diabetes (uncomplicated)	5 (7.0%)	171 (11.5%)	(*n* < 5)	16 (11.3%)
Diabetes with complications	(*n* < 5)	30 (2.0%)	(*n* < 5)	(*n* < 5)
Dementia	(*n* < 5)	40 (2.7%)	(*n* < 5)	(*n* < 5)
Cardiovascular disease	(*n* < 5)	17 (1.1%)	0 (0%)	(*n* < 5)
Hypertension	16 (22.5%)	328 (22.0%)	7 (17.5%)	26 (18.3%)
COPD	8 (11.3%)	143 (9.6%)	(*n* < 5)	14 (9.9%)
Heart failure	17 (23.9%)	383 (25.7%)	8 (20.0%)	27 (19.0%)
Renal transplant	0 (0.0%)	6 (0.4%)	0 (0%)	0 (0%)
Renal dialysis	0 (0.0%)	20 (1.3%)	0 (0%)	0 (0%)

^a^1492 episodes in 206 unique patients

*COPD*, chronic obstructive pulmonary disease; *eGFR*, estimated glomerular filtration rate; *IQR*, interquartile range; *SD*, standard deviation

### Propensity score matching

Before matching, the two groups had little overlap in their propensity scores, with median (interquartile range) of 0.49 (0.17, 0.84) for the alendronate users and below 0.001 (*<* 0.001, 0.003) for the non-users. After matching, the median (IQR) of the propensity scores were more similar, at 0.19 (0.08, 0.43) in the alendronate users and 0.13 (0.04, 0.43) in non-users.

**Fig. 2:**
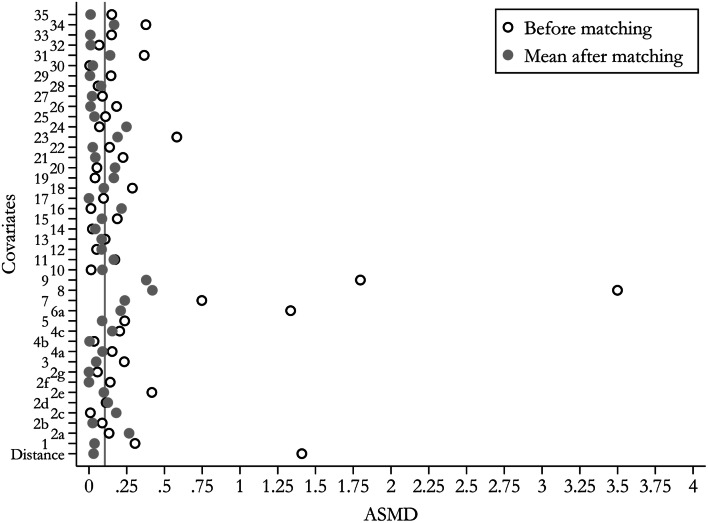
Covariate balance before and after matching usiung ASMD

Forty of the 71 alendronate users were matched to 142 non-users. Matching improved balance and reduced differences to acceptable limits (ASMD *<* 0.1) for most of the assessed participant characteristics included in the model (Fig. 2). However, relevant differences remained for key confounders, including baseline (index) femoral neck BMD (0.55 versus 0.62 gr/cm^2^), use of systemic glucocorticoids (27.5% versus 35.9%) and previous hip fracture history (12.5% versus 3.5%). Charlson comorbidity index, polypharmacy, number of hospital visits, history of key comorbid conditions (complicated diabetes, hypertension, chronic heart failure and COPD) and previous use of antiepileptics or antidiabetic therapies also still had unacceptable imbalances (ASMD *>* 0.1) after matching. Matching excluded more than 91% of the potentially eligible alendronate non-users.

### Annualized bone mineral density changes over time

**Fig. 3:**
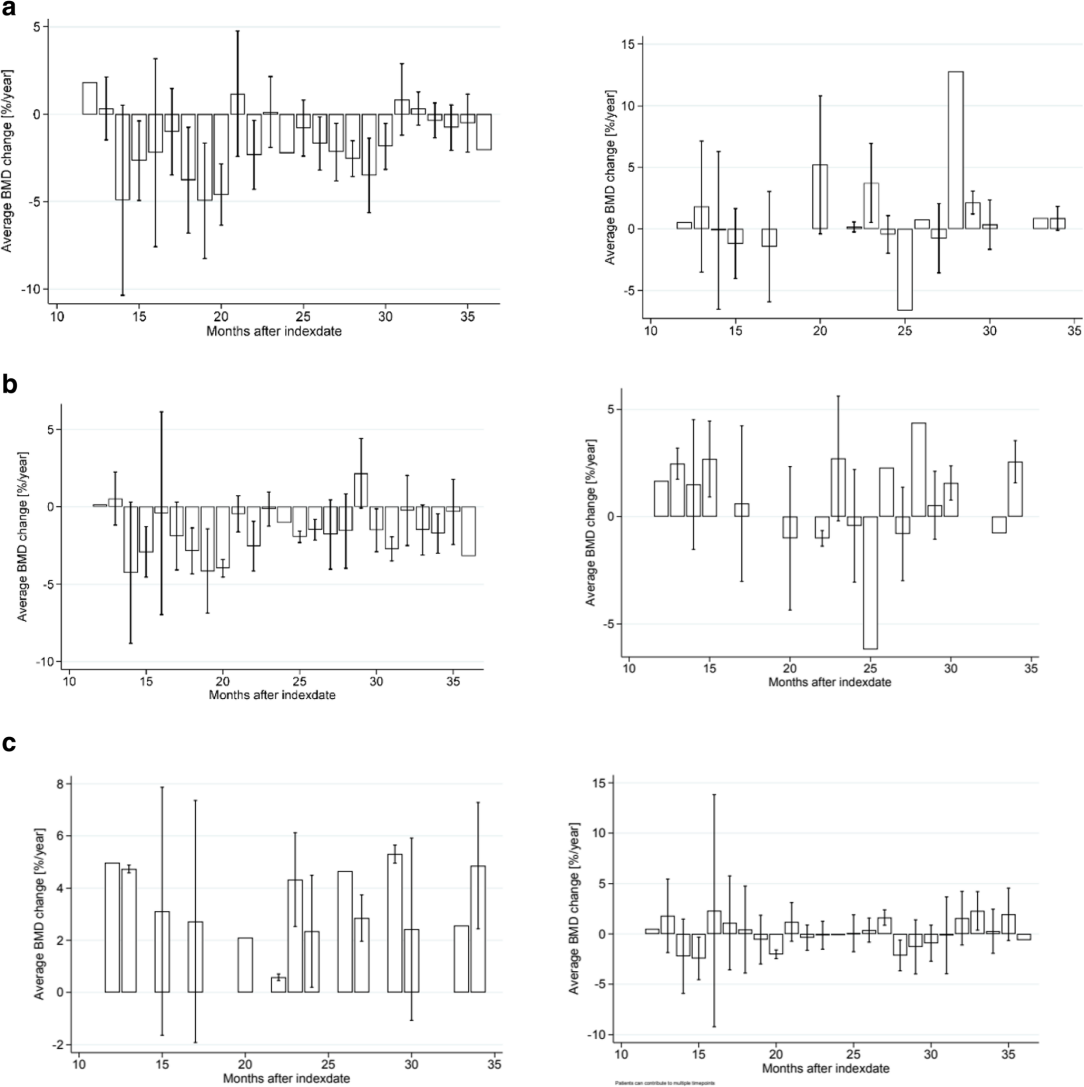
Average bone mineral density (BMD) change per month for bisphosphonate users (left) and non-users (right) in the femoral neck (**a**), hip (**b**) and spine (**c**)

In the propensity-matched cohorts (pre-specified primary analysis), alendronate non-users lost on average 1.59% of their baseline femoral neck BMD per year, whereas alendronate users increased their BMD by 1.07% per year of therapy. The average percentage BMD changes in the propensity-score-matched cohort are detailed in Table 3. In a linear regression model, after propensity score matching, the mean difference (95% CI) in percentage annual change in femoral neck BMD between alendronate users and non-users favoured the users by + 2.65% (1.32%, 3.99%) per year of alendronate exposure. Average femoral neck BMD change per month for alendronate non-users and users is shown in Fig. 3a.

**Table 3:** Results of the analysis of bone mineral density (BMD) changes in participants exposed and unexposed to bisphosphonates

	Analysis	Non-user		User		Mean difference (95% CI)
		*N*	BMD change	*N*	BMD change	
Femoral neck	PS-matched	142	− 1.59	40	1.07	2.65 (1.32, 3.99)
	Multivariable	1492	− 1.67	71	0.63	2.14 (1.22, 3.05)
	PS-adjusted	1492	− 1.67	71	0.63	2.15 (0.97, 3.34)
Spine	PS-matched	142	0.34	40	3.36	3.01 (1.74, 4.28)
	Multivariable	1492	0.65	71	3.98	2.14 (1.22, 3.05)
	PS-adjusted	1492	0.65	71	3.98	2.87 (1.62, 4.12)
Total hip	PS-matched	142	− 1.16	40	0.95	2.12 (0.98, 3.25)
	Multivariable	1492	− 2.14	71	0.82	2.29 (1.46, 3.11)
	PS-adjusted	1492	− 2.14	71	0.82	1.91 (0.82, 3.00)

*PS*, propensity score

In the analyses including the full cohort, alendronate non-users lost on average 1.67% of their baseline femoral neck BMD per year, whereas alendronate users gained an average 0.63% per year on treatment. The results of linear regression modelling supported the findings from the propensity-matched analysis, with a multivariable-adjusted beta coefficient (mean difference between groups) of + 2.14% (1.22%, 3.05%) and a propensity-score-adjusted beta of + 2.15% (0.97%, 3.34%) per year.

In the analysis of secondary outcomes, BMD at the spine increased amongst alendronate users by 3.98% per year for the full cohort and by 3.36% in the propensity-matched cohort. Alendronate non-users in the full cohort increased their BMD by 0.65% per year and in the matched cohort by 0.34% per year. The mean (95% CI) difference in percentage BMD change was again in favour of alendronate users, with betas of + 3.01% (1.74%, 4.28%) per year for the propensity-score-matched analysis, + 2.14% (1.22%, 3.05%) for the multivariable analysis and + 2.87% (1.62%, 4.12%) for the propensity-score-adjusted analysis. Average spine BMD change per month for alendronate non-users and users is shown in Fig. 3b.

Total hip BMD improved by an average of 0.82% per year for the full cohort of alendronate users and by 0.95% for the matched cohort. Alendronate non-users in the full cohort lost on average − 2.14% BMD per year and those in the propensity-matched cohort lost − 1.16% per year. The mean difference was + 2.12 (0.98%, 3.25%) per year in favour of alendronate users in the propensity-score-matched model, + 2.29% (1.46%, 3.11%) for the multivariable model and + 1.91% (0.82%, 3.00%) for the propensity-adjusted model. Average hip bone mineral density (BMD) change per month for alendronate non-users and users is shown in Fig. 3c.

The pre-specified sensitivity analysis using DXA measures taken between 3 and 5.5 years after index date could not be performed in the matched analysis as only 1 alendronate user and 12 non-users had valid follow-up data in this time window.

## Discussion

We used region-wide population-based data from Funen, Denmark. Despite the size of the source dataset, only 71 female participants were identified with stage 3B–5 CKD who were given alendronates, continued the treatment for at least 1 year and had at least two recorded BMD measurements. From around 200 eligible participants not using alendronates, only 1609 records of BMD measurements at different times were available. Propensity score matching led to the exclusion of almost half of the users and more than 90% of the non-users. Although matching helped to reduce confounding, imbalance remained when alendronate users and matched non-users were compared, including in key confounders such as baseline BMD, fracture history and use of systemic steroids. The small sample might demonstrate the low likelihood of Danish patients with stage 3B–5 CKD being prescribed alendronates, reflecting the safety concerns and lack of evidence on the efficacy that led to this study.

The primary propensity-matched cohort analysis identified a significant, clinically relevant effect, with a + 1.1% femoral neck BMD gain per year in alendronate users, compared with a − 1.6% bone loss at the same site per year in matched non-users. This is equivalent to a + 2.7% difference in BMD change per year in favour of alendronate users. The full cohort multivariable and propensity adjustment analyses found consistent results, with a + 2.1% and + 2.65% difference per year favouring alendronate use, respectively. Consistent results were found for the secondary outcomes. Lumbar spine and total hip BMD improved by 3.4% and 1.0% per year in alendronate users, compared with that by 0.3% and − 1.2% (bone loss) per year in the propensity-score-matched non-users, respectively. This equated to a significant + 3.0% per year average difference in the lumbar spine and a + 2.1% difference in the total hip, both favouring alendronate users. Multivariable and propensity-adjusted models found similar results.

### Limitations and strengths

This cohort analysis has limitations. The eligibility criteria excluded most of the records in the database including male patients and users of other oral bisphosphonates. Although this was not surprising, as bisphosphonates are to be used with caution in patients with stage 3 CKD and are contraindicated in stages 4 and 5, it is possible that the generalizability of the findings might have been compromised. The need for repeat DXA testing to obtain BMD change over time, the outcome of interest in this study, excluded more than half of the potentially eligible bisphosphonate users. These patients may have differed from the included participants, as BMD monitoring during treatment is not routine practice in actual care conditions. Current evidence suggests that routine monitoring of BMD within 3 years of starting bisphosphonates in postmenopausal women is clinically unhelpful1 (36) and may contribute to a lack of follow-up densitometry in an unexpectedly large proportion of those treated. Trials of bisphosphonates have shown a significant attenuation of BMD gain after the first 12 months of therapy (15, 37). The overlap between alendronate users and non-users was small, excluding almost half of the alendronate users and over 90% of the non-users when propensity score matching was applied. However, the multivariable and propensity adjustment models that include the full cohort gave reassuringly similar estimates to the propensity-matched analysis. Only women were included in these analyses, possibly related to the high rate of exclusion. The findings are thus limited to the female population.

This analysis also has some strengths. The dataset used has high granularity and quality and comprises information from a number of linked data sources. It is a unique data source for research as it includes comorbidity, hospital contacts, biochemistry, drug use, BMD testing and related questionnaires (38). The dataset also includes most of the population of a whole region of Denmark, thus maximizing the external validity of our findings.

The observed differences in femoral neck BMD in alendronate user versus non-user participants over 1 year were similar to those observed in trials of postmenopausal women (15). However, our alendronate users had only half the increase in BMD seen in the trial’s treatment arm after 1 year of therapy, where the increase in femoral neck BMD was 2% with alendronate (15). Although not statistically significant, the total hip is certainly the most relevant site of the upper extremity of the femur in the context of a follow-up with or without treatment. It is difficult to make generalization given the small size of our study in the findings. The difference at the spine in our alendronate users was also smaller than seen in the trial, where there was a 4.2% increase after 1 year. It is difficult to compare our unexposed CKD participants with those in the placebo arm of the trial as the trial participants who received the placebo also received calcium and vitamin D supplements.

Early BMD changes during therapy are difficult to interpret due to regression to the mean (39) with most patients who lose BMD in the first year of alendronate therapy going on to have significant increases in BMD in the second year. Our attempt to address this issue in a sensitivity analysis was inhibited due to a lack of power. The relationship between BMD change and fracture risk may differ in postmenopausal osteoporosis and bone fragility in CKD as they have different aetiopathology, with the potential involvement of adynamic bone disease in bone fragility in CKD (26). The disconnect between treatment-related BMD increases and fracture risk is exemplified in trials of fluoride therapy, where increased BMD was associated with a paradoxical increased risk of fracture (40). The artefactual effects of vascular calcification on spinal BMD further complicate the interpretation of BMD in patients with CKD (41).

## Conclusions

We demonstrated that alendronate therapy had a positive effect on BMD in female patients with stage 3B–5 CKD. BMD is the best surrogate available for bone strength in clinical practice, and RCTs using BMD as a primary outcome have been accepted by regulators as bridge studies to extend the indication of alendronate therapies for men (25) and for the prevention or treatment of steroid-induced osteoporosis (38). We found that BMD improved more (by 2% to 3% per year, depending on skeletal site) in alendronate users than non-users. In fact, BMD decreased over time when participants were off alendronate therapy, by about 1% per year in the femoral neck and total hip. The result had an average difference of + 2.7%, + 3% and + 2.1% BMD change per year in the femoral neck, lumbar spine and total hip, respectively, all in favour of alendronate users. These findings were not modified by age, previous fracture history or baseline renal function.
