# Correction to: Alendronate use and bone mineral density gains in women with moderate-severe (stages 3B–5) chronic kidney disease: an open cohort multivariable and propensity score analysis from Funen, Denmark

**DOI:** 10.1007/s11657-021-01001-9

**Published:** 2021-09-17

**Authors:** M. Sanni Ali, Martin Ernst, Danielle E. Robinson, Fergus Caskey, Nigel K. Arden, Yoav Ben-Shlomo, Mads Nybo, Katrine H. Rubin, Andrew Judge, Cyrus Cooper, M. K. Javaid, Anne P. Hermann, Daniel Prieto-Alhambra

**Affiliations:** 1 Faculty of Epidemiology and Population Health, Department of Non-Communicable Disease Epidemiology, London School of Hygiene and Tropical Medicine, London, UK; 2 Center for Statistics in Medicine, Nuffield Department of Orthopaedics, Rheumatology and Musculoskeletal Sciences, University of Oxford, Oxford, UK; 3 Department of Public Health, Saint Paul Hospital Millennium Medical College, Addis Ababa, Ethiopia; 4 OPEN, Department of Health, University of Southern Denmark, Odense, Denmark; 5 Department of Public Health, Clinical Pharmacology and Pharmacy, University of Southern Denmark, Odense, Denmark; 6 Population Health Sciences, Bristol Medical School, University of Bristol, Bristol, UK; 7 Nuffield Department of Orthopaedics, Rheumatology and Musculoskeletal Sciences, University of Oxford, Oxford, UK; 8 MRC Lifecourse Epidemiology Unit, Southampton, UK; 9 Department of Clinical Biochemistry and Pharmacology, Odense University Hospital, Odense, Denmark; 10 Department of Endocrinology, Odense University Hospital, Odense, Denmark; 11 GREMPAL Research Group (Idiap Jordi Gol Primary Care Research Institute) and CIBERFes, Universitat Autonoma de Barcelona, Barcelona, Spain

**Correction to: Archives of Osteoporosis (2020) 15: 81**

**https://doi.org/10.1007/s11657-020-00746-z**

The article *Alendronate use and bone mineral density gains in women with moderate-severe (stages 3B–5) chronic kidney disease: an open cohort multivariable and propensity score analysis from Funen, Denmark*, written by *M. Sanni Ali & Martin Ernst & Danielle E. Robinson & Fergus Caskey & Nigel K. Arden & Yoav Ben-Shlomo & Mads Nybo & Katrine H. Rubin & Andrew Judge & Cyrus Cooper & M. K. Javaid & Anne P. Hermann & Daniel Prieto-Alhambra*, was originally published Online First without Open Access. After publication in volume 15, issue 1, article number: 18 (2020) the author decided to opt for Open Choice and to make the article an Open Access publication. Therefore, the copyright of the article has been changed to © The Author(s) 2020 and the article is forthwith distributed under the terms of the Creative Commons Attribution 4.0 International License, which permits use, sharing, adaptation, distribution and reproduction in any medium or format, as long as you give appropriate credit to the original author(s) and the source, provide a link to the Creative Commons licence, and indicate if changes were made. The images or other third party material in this article are included in the article’s Creative Commons licence, unless indicated otherwise in a credit line to the material. If material is not included in the article’s Creative Commons licence and your intended use is not permitted by statutory regulation or exceeds the permitted use, you will need to obtain permission directly from the copyright holder.

To view a copy of this licence, visit http://creativecommons.org/licenses/by/4.0/

